# The Problem of Man's Origin

**Published:** 1935

**Authors:** A. Rendle Short

**Affiliations:** Professor of Surgery in the University of Bristol; Surgeon, Bristol Royal Infirmary


### The Bristol Medico-Chirurgical Journal

*" Scire est nescire, nisi id me
Scire alius sciret."*

SPRING, 1935.

### THE PROBLEM OF MAN'S ORIGIN.

The Presidential Address, delivered on 10th October, 1934, at the opening of the Sixty-second Session of the Bristol Medico-Chirurgical Society.

BY

A. Rendle Short, M.D., F.R.C.S.,

*Professor of Surgery in the University of Bristol ;
Surgeon, Bristol Royal Infirmary.*

In 1927, at a Presidential Address of the British Association, Sir Arthur Keith said that this problem could be regarded as definitely settled ; man is derived not, of course, from any living anthropoid ape such as the gorilla or chimpanzee, but from an ape-like ancestor. The common stem giving rise to man and the apes probably diverged in Miocene times, and our immediate ancestors were intermediate in structure between modern man and the ape. No doubt this view is very widely accepted, especially by the older anatomists and by the writers of orthodox text-books of science ; no doubt it is commonly taught in nearly every university in the world. Very many facts and

B

observations seem to confirm it. But Science has a way of upsetting our "settled conclusions" just when we are beginning to feel happy and secure about them, and I propose to bring evidence before you this evening, not to establish an alternative theory—apparently the time has not yet come for that to be done—but to show that though Sir Arthur Keith declared that Darwin's theory of the ascent of man would never be shaken, it is being shaken. Mr. Pyecraft, one of the Zoologists at the Natural History Museum, South Kensington, wrote recently concerning the Theory of Natural Selection generally: "We seem to be threatened with a recrudescence of the controversy over the Darwinian theory. But now the conflict is not to be between learned professors of biology on the one side and the Church and the people on the other, but an internecine warfare, that is to say, between ourselves. It has taken something like fifty years to secure what we might call orthodoxy among the elect; now all is to be thrown into the melting-pot again."

The same appears to be true with regard to the ape theory of man's origin. The doubts about it are beginning to percolate down to the newspapers. The *Morning Post* wrote, just about the time when England went off the Gold Standard: "There are disturbing signs that the scientific world may have to go off the ape standard. Speeches at last week's meeting of the British Association suggested that scientists are uncertain whether the stability of physical evolution can be maintained, and now Professor Sergio Sergi, at the World's Anthropological Congress, seems to be depressing the value of the 'missing link.' Owing to the general uneasiness that prevails, it is impossible to give authentic quotations for the evolution theory, but personally I am getting into something else as soon as I can!" And in a more serious vein, the *Daily Telegraph,* in a review of a book we shall presently be quoting from, said in December, 1933: "Since the first flush of enthusiasm which followed the enunciation of the Darwinian theory of evolution, the tempo of the science of anthropology has suffered a surprising slowing up. This branch of knowledge has advanced from certainty to perplexity." It may be said: "But this is only the opinion of newspaper men." We turn, therefore, to the scientists.

Let us begin by reviewing the evidence for the ape line of descent. The first and greatest argument, of course, is the very close anatomical similarity between the human body and that of the gorilla or chimpanzee. The likenesses are so numerous and so well known that it would be tedious and unnecessary to enumerate them, they are so obvious that in the opinion of many nothing more need be said; man and the ape must be brothers. Amongst animals bodily resemblances have generally been taken to prove blood-relationship. But there is another side to the matter. Although there are striking resemblances, there are also very constant differences. The human brain is far larger and more developed. The ape has a projecting muzzle, a hairy coat, and a foot quite unlike ours; the great toe is opposable like a thumb. Man has no vibrissæ, (tactile hairs); every other mammal has them. The apes have no hymen. No doubt it will be replied that these are merely the differences between species or genera, but a much more considerable point is next to be mentioned. The trend of modern zoological research goes to show that likeness of bodily structure is *no proof of common descent or blood-relationship.* There is a phenomenon amongst animals, living and extinct, known by the name of "Convergence"; two totally unrelated animals, widely different in their geological history and zoological relationships, may have a strangely similar bodily structure or individual organs, if their mode of life is similar. And this Convergence is not an occasional and exceptional phenomenon; examples of it are numerous and widespread. Let me remind you of a few. How like the common newt, that divides its time between stream and shore, is the crocodile, whose habits in that respect are similar! Yet the crocodile is a reptile and the newt an amphibian. Their zoological relationships are very far apart; their resemblance is due to the suitability of that particular pattern of legs, tail and general conformation for a life spent betwixt land and water. The spermatozoon of vertebrates, *e.g.* toad, is, down to minute details, like a free-swimming, lowly form of life called Trichomonas. But no one imagines that vertebrates are descended from Trichomonas. The extinct (Mesozoic) plants called Bennettitales show a sort of flower, with male and female elements and pollen, but they are Gymnosperms, allied to modern Cycads, and cannot possibly be ancestors of modern flowering plants.

Common wheat exists in several varieties, bearded and beardless; white, red or black eared; winter and spring. But just the same varieties are found of other wheats, spelt, rye and barley. This must be an inherent law of grain; it cannot be chance.

The Dipnoi (air-breathing fish living in mud or water) cannot be the ancestors of frogs, toads, etc., but they share with them the paired lungs, the partitioned auricle (of the heart) and many other characters.

The eyes of the octopus are just like those of a mammal, with cornea, iris, ciliary body, lens and retina; but the octopus is not the ancestor of the vertebrates. Lowly vertebrates have no eyes (amphioxus) or a very elementary eye (the hag fish). Two animals are known that have eyes like an old gentleman's bifocal spectacles, the upper half to see in air and the low in water, but one is a fish and the other is a beetle.

Three types of fish, the electric eel, Torpedo and Malapterurus, can give powerful electric shocks, but they are quite unrelated. The claws of a lobster and of a scorpion are on the same pattern. The glow-worm and the firefly, and also certain deep-sea fish, are luminous in the dark. One of the most remarkable examples of Convergence is furnished by the marsupials (pouched mammals of primitive type) of Australasia. There are forms that mimic most of the common types of the mammals of Europe, Asia and Africa. There is a volplaning opossum like a flying squirrel or flying lemur, the flesh-eating Thylacine like a wolf, another marsupial like a rat, and another like a bear! Nor is it only in outward form that Convergence is seen. The crocodile, like the bird, has a four-chambered heart. The extinct flying lizard, the pterosaur, had air-filled bones, and the foramen admitting the air situated just where it is in birds.

Bower points out that both plants and animals are bisexual, but it is scarcely credible that they have a bisexual common ancestor. Osborn calls attention to the strange parallelism between extinct reptiles and modern mammals; the huge dinosaurs with horns (Triceratops) like a rhinoceros; ichthyosaurus, like a whale; pterosaurs, like a bat; flesh-eating cynodonts with teeth like a dog; iguanodon, walking on its hind legs and tail like a kangaroo; the turtle, armour-plated like an armadillo, or the extinct glyptodon. Surely all this must be law, not chance. Especially when we find that each of these types require not one, but many coincident modifications; *e.g.* the heavy-headed rhinoceros must have massive legs and a strong neck; the flesh-eating Thylacine, the wolf and the extinct cynodont must have agility to hunt their prey.

The most recent, and one of the most eminent of writers on the descent of man is Professor Le Gros Clarke, who on the whole is in favour of the theory of descent from an ape ancestor, but he frankly acknowledges the difficulties and pitfalls of the hypothesis. He says: "In the evaluation of genetic affinities anatomical *differences* are more important as negative evidence than anatomical *resemblances* are as positive evidence. It becomes apparent that if this thesis is carried to a logical conclusion, it will necessarily demand a much greater scope for the phenomenon of parallelism or convergence in evolution than has usually been conceded by evolutionists. The fact is that the minute and detailed researches which have been carried out by comparative anatomists in recent years have made it certain that parallelism in evolutionary development has been proceeding on a large scale, and is no longer to be regarded as an incidental curiosity which has occurred sporadically in the course of evolution. Indeed, it is hardly possible for those who are not comparative anatomists to realize the fundamental part which this phenomenon has played in the evolutionary process."

We are driven to the conclusion, therefore, that the similarity between man and the ape may be another example of Convergence; in other words, the resemblances do not definitely prove blood-relationship.

But further, as Wood Jones, the Professor of Anatomy in the University of Melbourne, has pointed out, there are some anatomical features that make it easier to believe the apes are descended from man—an impossible hypothesis—rather than man from an extinct ape. The course of evolution never retraces its steps (Dollo's Law). If a modification has once been made, it persists. Now in some respects man's structure is more primitive than that of the apes. Like early mammals, but unlike the apes, he retains the ethmo-lachrymal, ethmo-sphenoid, and spheno-parietal articulations. The male external genitalia are more like those of primitive primates than those of the ape. Some primitive muscles, *e.g.* the pyramidalis and the pronator radii teres, are absent in the apes.

According to the law of Recapitulation, every animal has to climb up its own genealogical tree, that is to say, its ontogeny repeats its phylogeny, or its development in embyro gives evidence of its ancestry. Also, throw-backs may occur, that is, pathological specimens will be born from time to time that resemble the ancestor. Judged by either of these tests, the ape-ancestor theory stands definitely discredited. It is true the infant may have a hairy skin (lanugo), but so have nearly all mammals besides the apes. The sloped-back forehead, great eyebrow ridges, projecting muzzle, and opposable great toe are never seen in the human fœtus ; in fact, the ape fœtus is more like a human being than *vice versa.* The Darwinian tubercle on the human ear, and multiple nipples, which are often quoted as proving man's animal ancestry, are nothing to the point, because no ape has long, pointed ears or multiple nipples. Then consider the congenital abnormalities with which our profession makes us well acquainted : club foot, hare lip, cleft palate, congenital dislocations, nævi, supernumerary fingers or toes, spina bifida. None of these recall the ape. Who has ever seen a human with a projecting muzzle or opposable great toe? We come to the conclusion, then, that the argument from anatomy and development is too uncertain to be relied upon. In the opinion of Professor Wood Jones and others man's ancestor was not an ape, but must be sought much farther back, and in a much more primitive mammal. He suggests a little creature called Tarsius, which has been described as a living fossil.

The next main argument for the ape descent theory is derived from physiology. It is maintained, for instance, that ape's blood and human blood are identical, and differ from that of other mammals; this is taken to prove close relationship.

Far more work has been published on the comparative anatomy of the primates than on their comparative physiology. The best modern summary of the latter known to me is Zuckerman's *Functional Affinities of Man, Monkeys, and Apes*, published at the end of 1933. He shows that the blood of man and the apes cannot be regarded as identical. The blood of all animals has a good deal in common. The red corpuscles of man and most mammals are exactly alike under the microscope; the hæmoglobin of man and most mammals is indistinguishable by the chemist. As Nuttall showed in 1904, human serum and ape serum give the same precipitin reaction, though for ape's serum a much higher concentration is needed. But there are differences. Human blood contains hetero-agglutinins against the red corpuscles of the ape, and *vice versa*, so that it would be most dangerous to use ape's blood for transfusion in man. Basophile leucocytes, which are very scarce in human blood, are 3 per cent. of gorilla's white cells, 15 per cent. in the orang and 20 per cent. in the chimpanzee. By the use of anti-erythrocyte sera Landsteiner and Miller were able to show in 1925 that human and ape red-blood corpuscles are not identical and can be distinguished from one another, but those of a white man and a negro cannot. (This is very fairly pointed out by Sir Arthur Keith himself in his article on the origin of man in the last edition of the *Encyclopædia Britannica.*) They go on to conclude: "The experiments described show that a definite and constant serological difference is demonstrable between the bloods of man and the two anthropoids studied, chimpanzee and orang-outang"; and again: "This conclusion is in agreement with the accepted view that man has not evolved directly from any of the existing species of primates, as was formerly supposed, but that the Catarrhina, anthropoids and man have all sprung from a common stock."

Zuckerman further reminds us that man is physiologically different from the apes in his use of fire and tools, in his function of speech, his carnivorous diet and custom of monogamy. The last two he regards as related to one another. Anthropoid apes go about in small parties, one adult male with several females; they live mostly on a vegetable diet, and all find their own food. When man took to hunting for a living, the females, especially if pregnant or nursing, were less able to collect for themselves and had to be fed by the male, who could not protect all his wives from other marauding males, and did not want too many mouths to feed, so contented himself with monogamy.

We turn next to the evidence of palæontology. Here we must definitely put out of our minds the wholly imaginary picture of ape-men that appear from time to time in the illustrated London papers. As

Professor Wood Jones says: "The missing-link pictures must be deleted from our minds, and I find no occupation less worthy of the science of anthropology than the not unfashionable business of modelling, painting and drawing these nightmare products of imagination, and lending them in the process an utterly false value of apparent reality." He compares it with the pseudo-science of the old phrenological charts. Confining ourselves to real evidence, although the whole world has been ransacked in the search for "missing links," the actual discoveries have been few, and have taken unexpected forms. When the first skulls of Neanderthal man were found, with his huge brow ridges and head sunk on the chest, it seemed as if the true ape-man was before us. But Neanderthal man had nothing else ape-like about him. His brain was as big as ours; his teeth were truly human; he used tools, lit fires and buried his dead. So-called Rhodesian man appears to be closely allied to the Neanderthal type, and so does the Galilee skull. On the other hand, the Tauungs skull, called *Australopithecus*, also first described as a "missing link," is really that of a young anthropoid ape (Keith). A better case can be made out for three other fossil types, yet all with serious reservations. I append a very brief summary (the details are taken from the writings of Sir Arthur Keith). First in the field was *Pithecanthropus erectus*, found in 1894 at Trinil, in Java, by Dubois. These remains consist of the top of a skull, three teeth, and, found at a distance of some 15 yards, a femur. To these is to be added, possibly, a piece of a jaw. The beds in which these were found are considered to be late Pliocene, or more probably early Pleistocene. (Pleistocene means the Ice Age; Pliocene is the geological time-period next earlier.) The skull has been variously described as that of a large extinct ape (by Virchow, Bumuller, Kollman), or intermediate between man and ape (Dubois, Keith and others).

Next in order is *Eoanthropus dawsoni*, found by Mr. Charles Dawson at Piltdown in Sussex in 1911–1912. The geological level, again, may be late Pliocene or early Pleistocene, and again there is a discrepancy between the skull, which has the shape and brain capacity of modern man (Keith), and the jaw found near it, which is ape-like. A piece of worked elephant bone was also discovered close by.

More recently, in 1928–29, a nearly complete fossil skull with several fragmentary jaw bones and teeth has been found near Peking by Mr. Pei, and described by the late Dr. Davidson Black. These also are dated early Pleistocene. The skull has a brain capacity equal to that of a human, but is shaped rather like *Pithecanthropus*. The jaws and teeth, also, are intermediate between man and ape, so far as can be determined from the scanty nature of the evidence. The find is called *Sinanthropus pekingensis*. Worked flints with evidences of fire have been discovered in close association with the remains.

To sum up, there have undoubtedly been some strange types of mankind on the earth in prehistoric times, but that they link man with the ape is open to question.

It is clear that *Eoanthropus* was truly human ; it is possible, but not certain, that the jaw belonged to the same individual. They were not found close together. It is by no means so certain that the femur (human) and the cranium (ape-like) of *Pithecanthropus* have anything to do with one another. Peking man was truly human. Several "episodes" show how cautiously this palæontological evidence ought to be interpreted. In 1922 Professor Gregory, in America, found a single tooth which he thought was from a man-like ape, and called it *Hesperopithecus*—" the evening of the apes " ! The London papers, of course, came out with the usual imaginary drawings, half-ape, half-man. In 1927 it turned out that the tooth belonged to neither ape nor man, but to an extinct peccary ! In 1926, at Gardar, in Greenland, parts of a human skull and jaw were found, more ape-like in some respects than even the Rhodesian skull. It would have been a beautiful missing link, but for the fact that it came from a Norwegian twelfth-century Christian graveyard ! According to Professor Hansen, who described it, it is a throwback to primitive man. Sir A. Keith, with far greater probability, concluded that it is the result of acromegaly. But that raises the question whether the other abnormal skulls may not be due to disease also.

The real ape-like ancestor of man, therefore, remains to be discovered, if he ever existed. With this agree the candid words of Sir A. Keith, written in 1931 : " The fossil forms which represent this stage in the evolution of anthropoid and man have not yet been found ; their existence is inferred."

The most unexpected part of the palæontological evidence, however, remains to be mentioned ; the farther back we look for early man the more like ourselves he appears to be. When skulls with a cranial capacity equal to that of a modern European, and in all respects undeniable members of the species *Homo sapiens*, were discovered at Galley Hill, and at Calaveras in geological deposits at least as old as those in which *Pithecanthropus erectus* was found, it was felt that the evidence must be lying, and it was more or less discredited. But during the past year or two at Oldoway, Kanam, and Kanjera, in East Africa, Dr. L. Leakey has obtained skulls of the same great age, early Pleistocene, which are definitely modern in type, and associated with worked flints of human manufacture. These conclusions were verified last year by four committees of experts, anatomists and geologists, sitting simultaneously.

In 1925 a similar find was made in the City of London in digging the foundations for a building. We thus reach the surprising conclusion that *Homo sapiens* is as old as, or older than, any of his alleged ancestors, so far as at present discovered. In other words, the palæontological evidence reduces itself to something not far removed from nil. Reid Moir has found worked flints in East Anglia in earlier beds still, the Pliocene, which present evidences of the work of an intelligent people.

Very briefly, let us have a word with the psychologists. Some of them have been inclined to adopt the attitude that the ape at his best is as good as man at his worst. They emphasize the cleverness of the tricks which a chimpanzee may be taught, profess to be able to recognize ape language, and would have us believe that the Australian aboriginal or Central African native has barely the intelligence of a beast. But, as Zuckerman points out, it is very doubtful if, according to exact experiment, the chimpanzee is any more intelligent than a baboon, or, one might add, making due allowance for anatomical differences, a dog or a horse. And as for the African or Australian native, it is at length being recognized that you must not judge intelligence by that of the adult brought up in the wilds, but rather by that of the child given a proper education. Granted this, the best of the native children will be at least as good as the worst of the European.

Dr. Oliver in September, 1932, tested two large schools in Kenya, the one consisting of native boys and the other of the children of European settlers. He found that the average intelligence of the natives was only 85 per cent. that of the Europeans, but 14 per cent. of the natives surpassed the European average. It is noted that the Europeans were of good stock, probably higher than the average at home.

In assessing the relative brain power of Africans and Europeans, it must not be forgotten that the standard of bodily health in the white man is as a rule far better, and this is found to have an effect on learning capacity. Dr. J. H. Sequeira, in his admirable Chadwick Lecture of 28th April, 1932, drew attention to the astonishing multiplicity of diseases in the individual native, whose person in most instances presents the picture of a pathological museum. Thus in an investigation in one large district 94·8 per cent. of the children under 10 years of age showed symptoms of chronic malarial infection; 75 per cent. of the boys in a reformatory revealed infestation with hookworm; yaws is almost universal, and is a very disabling disease; syphilis is relatively unimportant; human tuberculosis, because of the absence of bovine infection, is less common than in Europe; pneumonia of pneumococcal origin is an especially fatal disease in natives and is very widespread. It is generally believed that the natives of Australia are as low in the scale of human intelligence as any, but an Australian aboriginal was good enough a year or two ago to play in first-class cricket; another is an eminent mathematician. Central African natives can be taught to make microscopic slides and find malarial parasites. To talk about the ape being as intelligent as man is too puerile to be taken seriously.

A curious experiment has lately been carried out by Professor and Mrs. Kellog of Indiana University. They brought up their own child, aged ten months, and a chimpanzee, aged seven and a half months, born in captivity, on exactly the same lines, down to the minutest details. The animal was fed upon a bottle, clothed, bathed, fondled and given exactly the same treatment as the baby. It was put in a perambulator and wheeled about, and in due course taught to walk and to feed itself with a spoon. Its mistakes were corrected, as one corrects the mistakes of a child. But the chimpanzee remained a chimpanzee and the child a child. It was definitely inferior in learning, though it was able to respond to 58 different words and the child to 68. It is put to the animal's credit that if hungry it would bite the professor's trousers! The experiment was brought to an end after nine months, that is to say, just when a child begins to make rapid strides in its education.

It is generally taken for granted that human intelligence shows a progressive development: that modern man is far cleverer than his Neolithic ancestors, and these again than the cave-man and the flint-chipper of Chellean (early Palæolithic) times. Of course, our civilization is immensely more complicated. Our machines and our medical skill would be a marvel to the ancient Britons. But the argument that therefore we have better brains is entirely fallacious. Other men have laboured and we have entered into their labours; other men have invented and observed, and we have learned what they had to teach. Some of the most remarkable of human discoveries were made so long ago that their origin is lost in the mists of antiquity. Who were the prehistoric geniuses who counted the days of the year, discovered the properties of opium, learned how to make cheese and soap, combined copper and tin into bronze, and invented the smelting of iron ore? Who made the first boomerang, or the first bow and arrow, or tamed the first horse? Indeed, we may push the inquiry farther back still, and pay tribute to the intelligence of the man who first chipped flints, and learned the secret of making fire. As Mr. Reid Moir, the great authority on Palæolithic man in East Anglia, has recently stated, the very earliest worked flints known to us, the pre-Chellean, present such differences that they must have been made by an intelligent and well-cultured people, who had, moreover, a great fight to maintain mastery over the numerous wild beasts that shared the lordship of the world with them. No wonder the earliest known skulls had brains as big as ours.

In this connection we may quote the words of Professor McDougall, of Harvard University, a leading authority on psychology: "It is now widely recognized that the strict neo-Darwinian theory of organic evolution is inadequate. . . . It finds itself, at the conclusion of its attempts, with mind upon its hands as an enormous remainder or surd, that cannot intelligibly be brought into the scheme, or ignored, save at the cost of the absurdity of the whole scheme."

Let us conclude with a few quotations from first-class authorities. Professor Le Gros Clarke, an advocate of the ape-descent theory, writes: "While, however, we may accept the thesis of man's descent from 'lower' forms of life, there is by no means a consensus of opinion among biologists as to the precise route by which the human family arrived at its present status, or what may have been the real nature of its immediate progenitor." In his opinion the common ancestor of man and the apes must have been very far back, and quite a small animal, no larger than a gibbon. It is the different structure of the foot that leads him to this conclusion. He further recognizes that no mere play of external forces upon a more primitive organism that reacts to its environment in obedience to what Darwin called Natural Selection is adequate to explain the origin of man.

With this accord the words of D'Arcy Thompson, the eminent zoologist, in his introduction to Berg's book on *Nomogenesis:* "How species are actually produced remains an unsolved riddle; it is a great mystery. Here at least is a conclusion that few men of our time will venture to dispute."

Professor H. F. Osborn, the greatest authority in America on fossil vertebrates, and head of the Natural History Museum, wrote: "Hence the idea of man's ape ancestry is a myth and a bogey, due to our previous ignorance of the real cause of human evolution." And again he writes of "the profound cleft between the ape and the man. It is our recent studies of the behaviourism of the anthropoid apes as contrasted with the behaviourism of the progenitors of man which compel us to separate the entire ape-stock very widely from the human stock."

Wood Jones comes to the following curious conclusion: "Man is more primitive than the monkeys and apes. . . . It follows that far from being a descendant of the apes, he may be looked upon as their ancestor. . . . Indeed, from the point of view of anatomy, I conceive it to be impossible to take any other view."

And Tilney, in his monumental work published two

C

or three years ago on *The Brain from Ape to Man,* says : " Apes are quite as unconcerned in the origin of man as they are innocent of participation in it."

I am well aware, disconcertingly aware, that the main trend of our line of investigation to-night has been destructive rather than constructive. To have to pull down a popular theory is less pleasing than to build up another with a wealth of new facts and argument. But we medical men have sufficient experience of the workings of living organisms to know that simple explanations generally prove to be inadequate. There remaineth very much land to be possessed.

